# Medical Opinion and Movement

**Published:** 1909-07-10

**Authors:** 


### Medical Opinion and Movement.

Various hypotheses have been conceived and formulated to explain the mechanism underlying the Treatment of Inflammatory Conditions by Bier's hyperæmic congestion. The most recent and most generally accepted is that of Sir Almroth Wright, which supposes that the hyperæmia induces a local increase in the immunising powers of the tissues or fluids. Attempts have accordingly been made to test this theory and to ascertain experimentally whether in conditions of hyperæmic congestion there is evidence of an increase in the immunising substances. Kleinberger, experimenting with rabbits, has studied the influence of passive congestion upon the production of agglutinins and hæmolysins. He confined his observations to these substances because they can be determined quantitatively and admit of control comparisons. As a result he finds that under the influences of passive congestion the formation of agglutinins is more rapid and also more abundant in comparison with animals not submitted to congestion with the elastic band. On the other hand, the formation of hæmolysins did not appear to be affected. According to the researches of Dr. Y. Shimodaira, however, the effect of passive congestion cannot be expressed in the augmentation of any particular kind of the multiple substances by which the organism reacts to infection, but is the result of the increased phagocytosis and augmentation of the bactericidal and antitoxic properties of the stasic fluids. This complexity explains, he thinks, the fact that in apparently identical conditions passive congestion is found in one case to be effectual, and in another quite ineffective.

---

Dr. H. Conradi, Director of the Bacteriological Institute at Neunkirchen, advocates the Sterilisation of Surgical Instruments, especially those of complex construction, in hot oil. He contends that the usual method of plunging them in boiling water containing 1 per cent. of carbonate of soda is not sufficient to ensure their complete sterilisation, as the spores of certain bacilli easily resist the action of boiling water. He recommends for this purpose the oil of sesame. The sterilising apparatus should be filled with this oil, and the instruments to be disinfected placed in it. Heat is then applied, and in the course of a few minutes a temperature of 200° C. or more is attained, which is sufficient to destroy all known varieties of spores. The steriliser can then be placed in a vessel of cold water, and the instruments in this way are quickly cooled so as to be ready for use. He points out that the sesame oil does not make the hands slippery, but supple, and offers therefore an additional advantage. As the boiling-point of this oil is between 310° and 325°, very little is lost in heating, and the same quantity can be used repeatedly. Moreover, this method of sterilisation is applicable not only to metallic instruments, but also to sounds, bougies, and catheters.

A discussion took place at a meeting of the Berlin Medical Society on the Serumtherapy for Diphtheria. Dr. Fritz Meyer advocated the administration of large doses of the serum as early as possible in the course of the disease, basing his views on experiments with animals, in which it was clearly shown that the serum if injected in sufficiently large doses is able to save the animals even when subjected to doses of the toxin many times mortal. The injections should be made either intramuscularly or intravenously, and for this purpose the serum should not be carbolised. The serum is of no use for conditions of cardiac weakness. For this purpose resort should be made to adrenalin. It was generally admitted that for ordinary cases intramuscular injections of serum suffice, but that in severe cases the intravenous method should be used, in order to secure an immediate effect without loss of time. Subcutaneous injections should be altogether abandoned, as it has been shown that it takes two or three days after such an injection before the antitoxin reaches its maximum strength in the blood. Consequently a serious loss of valuable time is involved. According to Dr. Morgenroth this loss of time can be reduced to an eighth by intramuscular injection.

---

The following case of acute cerebrospinal meningitis in which the most active and energetic measures led to a successful issue is reported in *Le Progrès Médical* by Adjutant-Major Dr. S. Heyraud of the 6th Infantry Regiment at Saintes. The patient, a soldier, was received into hospital on the night of April 19th, 1909, with a temperature of 39.9° C. He became much worse during the night, and the doctor was sent for early the following morning. He was then found to be unconscious, with the head drawn back, the back arched, and the legs flexed. He was very agitated and restless, and the patellar reflexes absent. The bladder was distended and micturition suspended. A diagnosis of meningitis was made and lumbar puncture performed. Thirty to 40 c.c. of purulent liquid were withdrawn and sent to the laboratory for examination. Immediately 15 c.c. of anti-meningococcal serum were injected intraspinally, and 25 c.c. subcutaneously; 2.5 c.c. of electrargol were also injected intravenously and 7.5 c.c. subcutaneously. A further subcutaneous injection of 10 c.c. of electrargol was made in the evening. Baths at 40° C. were given every three or four hours. The patient remained unconscious throughout the day, and the bladder was evacuated by catheter. The following day consciousness gradually returned. Lumbar puncture again gave 40 c.c. of purulent fluid. A further intraspinal injection of 20 c.c. of serum, and intravenous and subcutaneous injections of electrargol formed the second day's treatment, together with an injection of .05 c.c. of spartein sulphate. Marked amelioration was shown the next day, and lumbar puncture gave 5 c.c. of almost clear fluid. The injections of electrargol and spartein and the baths were continued. Under this treatment the condition of the patient steadily improved, and on the eleventh day the temperature fell to normal, and the patient rapidly became convalescent. Examination of the spinal fluid showed the presence of Weichselbaum's meningococci in large quantities. Inasmuch as the case was evidently of the fulminating type, Dr. Heyraud seems fully justified in the opinion that the recovery of his patient was entirely due to the energetic treatment he pursued.

THE perplexing condition known as "essential" hæmaturia, nephralgia, and other synonyms, is considered in two papers by Schwyzer and Pilcher in the *Annals of Surgery*. The former reports five cases in which an exploratory nephrotomy, done for renal hæmorrhage and pain, revealed no macroscopic evidence of disease, and was followed by complete cure of the symptoms: the cases have all been followed for periods of time ranging from four and a half years upwards, and there is therefore ample reason to regard the cures as permanent. This author emphasises the importance of taking every means of establishing the diagnosis—or rather of excluding every gross lesion: he also notes especially the great frequency with which trauma plays a part in the ætiology. The microscopic picture of portions removed for examination may show signs of severe kidney irritation, or nothing but blood. Renal colic, he says, with the most disquieting microscopic appearance of the sediment in the urine of one side can, after operation, disappear completely—the colic never to return, the urine to remain ideally cleared up. It is also of importance to exclude the possibility of the hæmorrhagic diathesis before suggesting operative measures on any patient suffering from renal hæmorrhage.

PILCHER, on the other hand, lays stress on the painless nature of "essential" hæmaturia and its dissociation from traumatic influences. He describes the condition as nearly always unilateral, and reports two cases of his own in which the lesion found was merely a varix of the veins of the kidney. He ascribes the cure which follows exploratory nephrotomy in these cases to the section of the veins affected: the operation is said to accomplish exactly what that of multiple ligature does in the treatment of varicosities in other portions of the body. Six of the main collecting venous radicles of the kidney are severed and permanently closed, and the course of the blood-stream is instantly changed to the anastomoses at the upper and lower poles of the kidney. He is sure that this angiomatous condition of the papillæ renales is a distinct pathological entity, resembling varicocele, hæmorrhoids, and varicose veins of the leg; and, further, that many of the cases of essential hæmaturia are instances thereof. Nephrotomy is the operation of choice, though decapsulation and fixation cure some cases. Nephrectomy is indicated only when a rapid and bloodless operation is essential, or when nephrotomy has failed to cure.

DR. LEONARD WILLIAMS, whose criticisms of the adenoid hypothesis of Nocturnal Enuresis and advocacy of Thyroid Medication for this complaint were referred to in the issue of May 15, p. 174, expands his views on comparative hypothyrea in the *British Journal of Children's Diseases*. Not only does Dr. Williams not believe that adenoids have anything to do with enuresis, but he does not even admit that they play a causal part in the production of the arched palate and other ingredients of the adenoid facies. He attributes these deformities to disordered calcification of the bones due to insufficient development and activity of the thyroid gland, and quotes expert laryngologists in support of his argument that removal of adenoids often fails to benefit palatal and dental irregularities. The patients exhibit also, in a majority of cases, a persistently subnormal temperature, and have often cold extremities, and fingers that "go dead." This symptom also is attributed to relative athyrea, for it disappears, together with the nocturnal enuresis, when thyroid extract is administered. It is also emphasised that the children are nearly always undersized, and weigh considerably less than the average for their age; this also is remedied very rapidly under treatment. The author concludes with some remarks on the signs of intolerance and overdosage by thyroid extract. The most constant is nasal catarrh, which appears very constantly, and long before tachycardia is noticeable. Another sign is the rising temperature and intolerance of heat on the part of the patient. As long as there is gain in weight the remedy is doing good, but as soon as there is a definite fall the drug should be immediately suspended. Dr. Williams' theories will no doubt be thoroughly tested by independent observers, and if his conclusions should be confirmed a notable advance in organo-therapy will certainly have been made.

ANOTHER aspect of Thyroid Insufficiency is dealt with in the *Bristol Medico-Chirurgical Journal* by Dr. D. A. Alexander, who reports the puerperal history of a woman who had been cured of myxœdema by thyroid extract. The patient was a normal child up to the age of about ten years, when she developed signs of thyroid insufficiency which were never properly treated until four years later. She was then taken in hand by Dr. Byrom Bramwell, and under thyroid medication did well. She still occasionally took the extract whenever dryness of the skin warned her of the possibility of relapse, and at twenty she married. Her first three pregnancies terminated successfully; and the fourth promised to do the same, for the urine was normal, emesis not excessive, and the general condition so good that no thyroid extract was taken. At the beginning of labour there was a profuse accidental hæmorrhage, but otherwise labour was easy. During the first week of the puerperium the patient expressed a desire for thyroid treatment, and the temperature oscillated about one degree per day. On the eighth night delirium was reported, and on the ninth a rigor with a temperature of 105° F.

Jaundice, thrombosis, and metastatic abscesses rapidly developed, and on the fourteenth day she died; autopsy was refused. It is suggested as a possible explanation of the case that during pregnancy the fœtal thyroid may compensate the mother for the inactivity of her own. Halsted proved that in pregnant bitches the thyroid can be almost exterminated without causing myxœdema. And it might conceivably follow that if such had happened in this case, acute symptoms of myxœdema might ensue on the completion of pregnancy. There was, however, no thyroid enlargement palpable in the infant, and the account given of the course of the case suggests a septic rather than a myxœdemic origin for the fatal complications which ensued.

Acute Pulmonary Œdema is recognised as an occasional complication of pregnancy. It may occur during labour, but is more frequently observed at the time of delivery, while occasionally it arises during the puerperium. Bonnet-Laborderie has recently attempted to solve the problem of its pathology. Huchard attributes it to the effect on the cardio-pulmonary plexus of a condition of aortitis, or peri-aortitis, but Pouliot has negatived this, at least as regards obstetrics. Clinical evidence has so far failed to trace it to hyperactivity of the suprarenal bodies, despite the fact that injections of adrenalin have produced the condition in rabbits. Most authorities agree that cardiac and renal affections are common antecedents, but cases also occur in which no organic lesions are found. The author believes that cases of this sort are the result of the increased blood-pressure which undoubtedly occurs during pregnancy, and is manifested equally in the lungs and in the rest of the body. The expulsive efforts of labour still further increase this pressure, and may end in acute dilatation of the heart. Moreover, evacuation of the uterus fails to relieve this hypertension.

The investigation of the micro-organisms most commonly concerned in secondary infections in Pulmonary Tuberculosis, a subject in which much interest is now being shown, is dealt with in the second annual report of the King Edward VII. Sanatorium, Midhurst. Twenty-three patients in all were examined. Of streptococci forty-two strains were obtained, very few of them either giving the typical reactions of streptococcus pyogenes, or belonging to the S. fæculis group. The majority of the staphylococci isolated were S. albus, there being only one specimen each of the aureus and citrens form. Three Gram-positive tetracocci organisms were isolated, and also a bacillus closely resembling the bacillus influenza. It is stated that only four varieties of bacilli were found. Three of the patients are under treatment with mixed vaccines of the various organisms, the opsonic index to one of the organisms being used as a guide in the determination of the correct time to administer the vaccine, and of the amount of the dose. After a course of this treatment tuberculin is then tried. The patients treated in this way have been advanced cases and all pyrexial. It has not been considered advisable to draw conclusions owing to as yet insufficient experience.

Should experience prove that it is as efficacious as it is said to be, the formaldehyde-sheet method of disinfecting rooms should prove a boon both to medical men and to parents, because it is so simple. It appears that many reputed formalin methods fail because the formaldehyde becomes polymerised as it is given off. This polymerisation is largely prevented, however, when phenol vapour is mixed with that of formalin. The following mixture is easily made: Formaldehyde solution (40 per cent), 3 parts; Phenol, 1 part. Of this mixture, about 8 oz. suffice for every 1,000 cub. feet of room volume. A sheet is moistened with the disinfectant and hung up in the room; an ordinary sheet will take about 4 oz. of the liquid, so that several will doubtless be required. The room is then left closed for at least two hours.

Acute Deficiency of Suprarenal Action in Infective Maladies is a condition that is being discussed a good deal in France just now, particularly by Dr. Emile Sergent. There may or may not be truth in his views; at any rate they are new, and well worthy of consideration. We know how cloudy swelling and even fatty degeneration may occur in all sorts of organs in severe infective maladies, such as diphtheria, typhoid fever, scarlatina, and so on. The results of such changes in the heart muscle are familiar; but there are not many who have even thought what may be the effects of similar changes in the cells of the suprarenal capsules. Dr. Sergent says that acute changes in the latter are responsible for at least a part of the general depression, asthenia, arterial hypotension, and hypothermia that may ensue after many of the infective diseases. Without necessarily agreeing with him, there seems to be no reason against a trial of suprarenal extract in these cases, not only when the symptoms have actually arisen, but even earlier in severe cases.

Insusceptibility to Vaccination is a subject upon which there are wide differences of opinion, some observers allowing it to be not uncommon, others almost denying its existence. That the ineffectiveness of vaccination in certain children depends more upon the lymph used or upon the vaccinator than upon any innate insusceptibility on the part of the child is strongly brought out by Dr. Leslie Thorne Thorne's report upon vaccination in the Medical Officer's supplement to the thirty-seventh annual report of the Local Government Board. The Animal Vaccine Establishment ceased its work in 1908, public vaccination with glycerinated calf lymph having rendered its continuance unnecessary. The establishment in question came into being in 1881, and a case of "insusceptibility" was never experienced at it by the different operators who had worked there during its existence of twenty-seven years, although 125,566 consecutive primary vaccinations were performed in that time. It seems clear that "insusceptibility" in the legal sense, that is to say, failure to vaccinate a child after three attempts, must in actual practice be an extremely rare event when vaccination is performed properly with reliable lymph.

